# Non-invasive sampling of water-borne hormones demonstrates individual consistency of the cortisol response to stress in laboratory zebrafish (*Danio rerio*)

**DOI:** 10.1038/s41598-022-10274-0

**Published:** 2022-04-15

**Authors:** H. L. E. Midttun, Ø. Øverli, C. Tudorache, I Mayer, I. B. Johansen

**Affiliations:** 1 Department of Paraclinical Sciences, Faculty of Veterinary Medicine, Norwegian University of Life Sciences, Ås, Norway; 2 Department of Preclinical Sciences and Pathology, Faculty of Veterinary Medicine, Norwegian University of Life Sciences, Ås, Norway; 3 Institute for Biology, Leiden University, Leiden, The Netherlands; 4 Department of Production Animal Clinical Sciences, Faculty of Veterinary Medicine, Norwegian University of Life Sciences, Ås, Norway

**Keywords:** Biological techniques, Physiology, Endocrinology

## Abstract

Glucocorticoid (GC) stress hormones are well-known for their impact on phenotypic traits ranging from immune function to behaviour and cognition. For that reason, consistent aspects of an individual’s physiological stress response (i.e. GC responsiveness) can predict major elements of life-history trajectory. Zebrafish (*Danio rerio*) emerge as a promising model to study such consistent trait correlations, including the development of individual stress coping styles, i.e. consistent associations between physiological and behavioral traits. However, consistency in GC responsiveness of this popular animal model remains to be confirmed. Such a study has so far been hampered by the small-bodied nature and insufficient blood volume of this species to provide repeated measurements of circulating GCs. Here, we adopted a technique that allows for repeated, non-invasive sampling of individual zebrafish by quantifying GCs from holding water. Our findings indicate consistency of the magnitude of post-stress GC production over several consecutive stress events in zebrafish. Moreover, water-borne GCs reflect individual variation in GC responsiveness with the strongest consistency seen in males.

## Introduction

Throughout the vertebrate lineage, glucocorticoid “stress hormones” (GCs, mainly cortisol and corticosterone) are central in shaping major aspects of individual phenotype. Traits under GC control include immune function, reproductive investment, reallocation of energy away from somatic growth towards stress coping, and various aspects of behaviour, neural plasticity and cognition^1–13^. Numerous studies in different animal models have revealed individual consistency and a degree of heritability of the magnitude of the GC response to stress (e.g.^14–18^). In other words, individuals tend to respond to episodes of stress with the same magnitude of the GC response and this tendency is passed on to their offspring. Furthermore, in a range of animal species and taxa (e.g. teleosts, rodents, birds and pigs), physiological and behavioral responses to stress are associated in such a manner that distinct stress coping styles along a reactive-proactive continuum can be identified^19–24^. Various terms are used to categorize individuals employing consistently different reaction norms in response to changes in the environment, but when physiological correlates (e.g. GC responsiveness) of consistent behavioral patterns are concerned, animals are commonly classified as either “reactive” or “proactive” based on their distribution along a shy-bold continuum (for reviews, see^19,24–26^). Stress coping styles are thus defined as a set of behavioral and physiological responses to stress that are consistently employed by one individual across unrelated and temporally separated situations^25^. Reactive individuals are characterized by having a high post-stress GC production whereas proactive individuals respond to stress with low GC production^22^. Ultimate and proximate mechanisms underlying such consistent trait correlations are probably complex and remain largely unknown. To understand these intricate mechanisms, we need novel model systems allowing the use of advanced molecular-genetic tools.

Among the vertebrate lineage, teleost fish (e.g. salmonids, zebrafish; *Danio rerio*, medaka; *Oryzias latipes*, goldfish; *Carassius auratus*) are now rapidly complementing or replacing rodent models in scientific disciplines like neurobiology, toxicology and immunology. In particular, the zebrafish is becoming an increasingly popular laboratory animal model in neurobiological and behavioural studies^27^. Considering the rapid development of behavioral screening^28–30^ and molecular genetic tools^31^ available for this species, zebrafish emerge as a promising model to reveal mechanisms underlying consistent phenotypic trait correlations. Several studies have demonstrated a strong covariation between behaviour and post-stress cortisol levels in zebrafish^22,32^. Moreover, distinct behavioral patterns appear to be generally consistent across contexts and time and influenced by selective pressures in zebrafish^33–35^.

In view of the above, covariation in behaviour and GCs and consistency in behavior are indicative of stress coping styles in zebrafish. However, to comply with the strict definition of stress coping style^25^, it remains to be determined whether physiological stress responsiveness (i.e. GC responsiveness) is a consistent trait in this species. Such a study has been hampered by the fact that zebrafish are small-bodied animals with blood volumes insufficient to provide repeated measurements of circulating GCs. Instead, whole-body GC levels have been used for several decades to assess the stress response of zebrafish^36–38^, like in other small-bodied fish such as young salmonids^39^ and flatfish^40^. These GC measurements are however terminal, without the possibility of repetition over time. This constraint has thus entailed a significant knowledge gap regarding individual consistency of the stress response, since techniques that allow for repeated, non-invasive sampling from in the same individuals across conditions are essential in this context^41^.

For teleost fish, a solution may be provided by extracting GCs from the fish holding water. In aquatic organisms, circulating steroid hormones are continuously released by the organism into the surrounding water. For example, Félix et al.^42^ validated that GC levels in the holding water reliably reflect baseline (i.e. resting levels) circulating plasma hormone levels in zebrafish. White et al.^43^ also demonstrated a strong positive correlation between zebrafish post stress holding water and whole-body cortisol in male zebrafish (2017), indicating that GC levels in holding water may accurately reflect post stress GC production in the body. This technique should therefore allow for non-invasive repeated measures of GC responsiveness from the same individual. Of note, it remains to be tested whether the technique also yields sufficient precision to assess GC variability in female zebrafish, where post stress cortisol production is considerably lower than in males^22,44,45^.

By repeatedly measuring post stress cortisol levels (the dominating GC in zebrafish) non-invasively, we show that GC responsiveness is a consistent trait in male and female zebrafish. In addition, water cortisol levels closely correlated to whole-body cortisol measured terminally although this association reached statistical significance only in male fish. Thus, our study corroborates that GC levels in holding water can be used as a proxy of post stress GC production at least in male zebrafish. The consistency of GC responsiveness in this animal model allows for future generation of zebrafish strains differing in stress reactivity that can serve as valuable models for studying the biology underlying stress coping styles, including individual vulnerability to stress-related diseases.

## Materials and methods

### Ethics statement

Zebrafish were maintained and handled according to the guidelines from the Zebrafish Information Network (ZFIN, http://zfin.org). All experimental protocols were approved by the Norwegian Animal Research Authority (NARA), following the Norwegian laws and regulations controlling experiments and procedures on live animals in Norway (permit number 11241). The study was carried out in compliance with the ARRIVE guidelines.

### Animals and housing

A total of 25 female and 24 male adult AB wild-type zebrafish, obtained from the Department of Production Animal Clinical Sciences, the Norwegian University of Life Sciences (Oslo, Norway), were housed in a recirculating Z Mod system (Marine Biotech), at a 14:10 light:dark cycle. Water temperature was kept at 28 °C, pH and conductivity were maintained at 7.4–7.6 and 500 µS respectively as according to the Zebrafish International Resource Center guidelines^46^. Fish were fed twice daily with commercial pelleted food (Special Diets Service, Witham, Great Britain) and twice with *Artemia nauplii* (Sep-art, Ocean Nutrition, Belgium). Prior to experiments, fish were starved for at least 12 h. Females and males were kept in separate tanks at a recommended density of 5 fish/L^47^.

### VIE-tagging

All fish were anaesthetized in 0.02% tricaine methanesulfonate (MS-222; Sigma, St. Louis Missouri, USA). Fish were subsequently injected subcutaneously with Fluorescing Visible Implant Elastomer (VIE) tags (Northwest Marine Technology Inc., Shaw Island Washington, USA) following the method by^48^, either adjacent to the dorsal fin, at the abdomen, or both, creating individually identifiable patterns. Immediately after recovery from surgery, fish were transferred to a holding tank containing clean system water. Testing was commenced after a minimum of 10 days recovery period. All fish were regularly visually examined for infections and open wounds during the recovery period. All individuals had healed completely before the start of testing.

### Stress testing and collection of holding water for cortisol analysis

**Figure 1:**
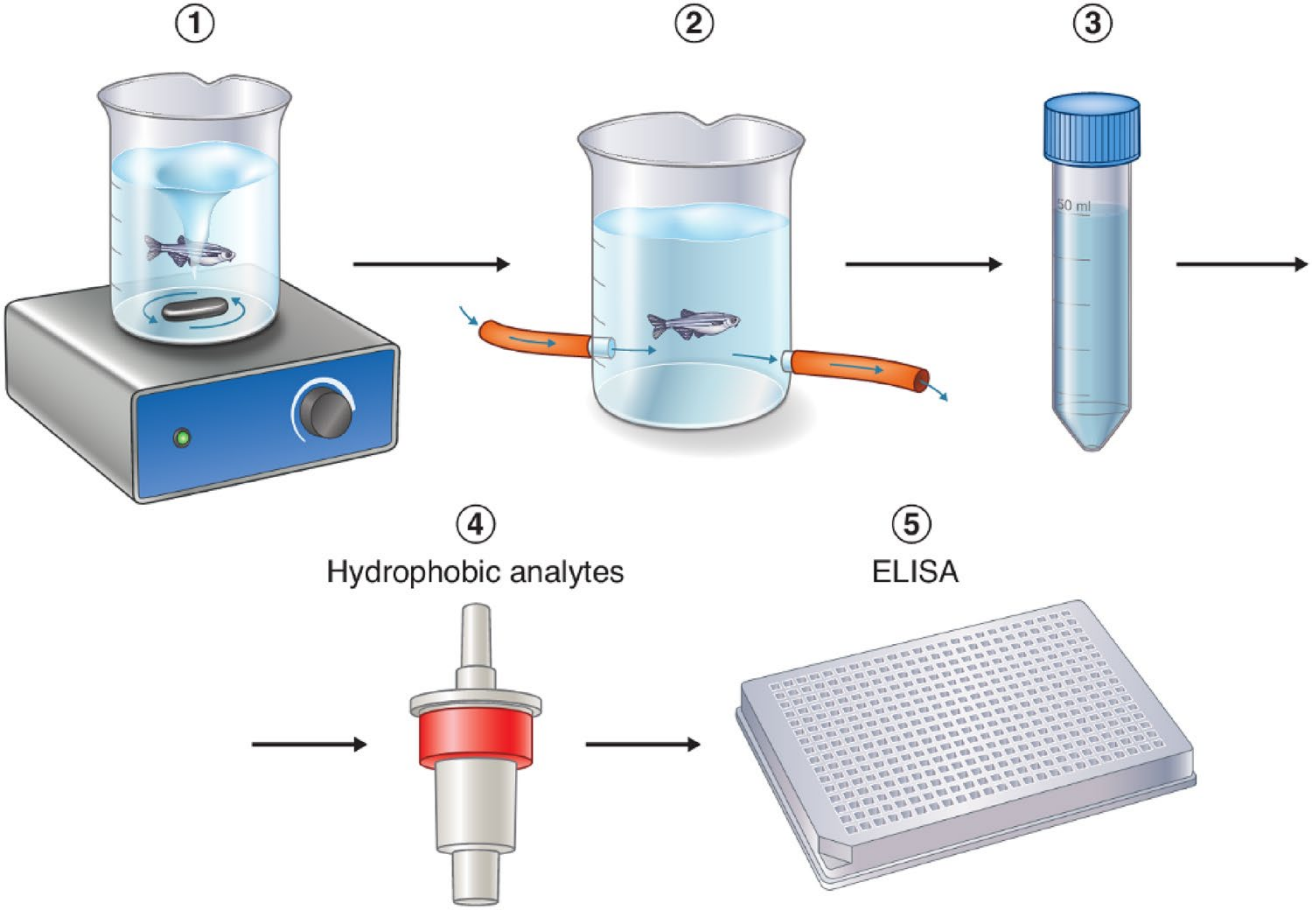
Experimental and analytical protocol for analysis of water-borne cortisol. (1) Fish were stressed in a glass beaker containing a stirring magnet and placed on a magnetic stirrer running at 100 RPM (2) before transferred to a plastic beaker connected to a flow-in and flow-out tube where water flow was shut off to allow cortisol to exit into the water. (3) Outflow water was then collected in 50 mL Falcon tubes (4) and cortisol extracted using C18 solid-phase extraction cartridges. (5) Water cortisol extractions were assayed using a commercial ELISA kit.

Fish were subjected to four consecutive stress tests in total separated by a minimum of 6 days, and between 08:00 and 11:00 to control for diel fluctuations in cortisol. The experimental and analytical protocols are illustrated in Fig. 1, with details following below. Initially, fish were individually netted from the holding tanks and transferred to a 250 mL glass beaker filled with 50 mL of system water containing a stirring magnet and placed on a magnetic stirrer. A UV flashlight (Northwest Marine Technology Inc., Shaw Island Washington, USA) was used to identify the fish before the stress test was initiated: the stirrer (VWR, Radnor Pennsylvania, USA) was set at the lowest stirring speed (100 RPM) and kept at this speed for 20 min. This speed was sufficient to induce constant swimming behaviour, interrupted by periods of burst-and-coast behaviour. After the forced swimming period, fish were netted to a 100 mL plastic beaker (VWR, Radnor Pennsylvania, USA) containing 50 mL of system water. The beaker was connected to a flow-in and flow-out tube, with the flow-in tube connected to system water through a valve. This flow-through system was set at a slow, constant flow of 0.3 L/min during the first 15 min after the stress test was finalized to capture the recovery phase of cortisol production rather than the build-up phase where the first is subject to greater individual variation compared to the latter^49^. The flow-through was then shut off and the fish was kept in the beaker for 1 h to allow cortisol to exit and accumulate into the water. Fish were then transferred back to their holding tanks until subsequent stress tests. The water was then collected in 50 mL Falcon tubes (Corning, Corning New York, USA) and kept at − 20 °C for a later analysis of cortisol concentration. In addition, two blank water samples were taken to account for residual cortisol concentrations in the system water used for stress testing (average 0.90 pg/ml) and average blank samples were subtracted from experimental samples before analyses. Following the 4th consecutive stress test, fish were immediately euthanized in an overdose of tricaine methanesulfonate (1 g/L, Sigma, St. Louis Missouri, USA)^50^. Fish were then weighed, and fork length was measured before they were frozen on dry ice. All fish were kept at − 80 °C until further analysis of whole-body cortisol.

### Cortisol extraction

Cortisol was extracted from water using C18 solid-phase extraction cartridges (Waters, Milford Massachusetts, USA). Cartridges were activated with 5 mL methanol and rinsed with 10 mL MQ water. Water samples were thawed at room temperature before being pumped through the cartridges by a peristaltic pump at 10 ml/min for 5 min (SCI-Q 323, Watson-Marlow, Falmouth, Great Britain). Cartridges were then rinsed with 10 mL MQ water using a syringe and all excess water was expelled by pressing air through the cartridge, before storing the cartridge at − 20 °C until further use.

Cartridges were thawed at room temperature before elution with 10 mL methanol into glass test tubes (130 × 16 mm, VWR, Radnor Pennsylvania, USA). Methanol was evaporated in an evaporator (Reacti-Therm III heater, Thermo Scientific, Waltham Massachusetts, USA) at 25 °C in a fume hood. Steroid hormones were reconstituted in 500 μL assay buffer supplied in the ELISA kit (DetectX®, Arbor Assays, Ann Arbor Michigan, USA). Test tubes were vortexed for 30 s, left on ice for 30 min, vortexed again and samples were transferred to microcentrifuge tubes (Eppendorf, Hamburg, Germany). All samples were stored at − 20 °C until further analysis.

Cortisol extraction from whole-body was carried out according to Ramsay et al.[^37^] with few modifications. Nine male and eight female fish were used for the extraction. Briefly, fish were thawed and transferred to a 5 mL microtube (Eppendorf, Hamburg, Germany) with 750 μL MQ water, the fish were homogenized using a handheld homogenizer (VDI 12, VWR, Radnor Pennsylvania, USA) for 90–120 s. The homogenizer was cleaned with MQ and ethanol between samples. After homogenization 4 mL of diethyl ether was added, before the samples were vortexed vigorously and poured into glass test tubes. An additional 4 mL of diethyl ether was added to the test tubes before they were vortexed for 30 s and put on ice. The samples were subsequently centrifuged for 2 min at 1320 g and stored at – 80 °C for 10 min to freeze the tissue so that supernatant containing extracted cortisol could be transferred to a new test tube. Following freezing, the diethyl ether was evaporated in an evaporator (Reacti-Therm III heater, Thermo Scientific, Waltham Massachusetts, USA) at 25 °C in a fume hood and extracted steroid hormones were reconstituted in 1 mL of assay buffer supplied in the ELISA kit (DetectX®, Arbor Assays). All samples were stored at − 20 °C until further use.

### ELISA

Whole-body and water cortisol extractions were assayed using a commercial ELISA kit (Arbor Assays, Ann Arbor Michigan, USA). Whole-body cortisol extractions were diluted 1:25 while water cortisol extractions were diluted 1:5 both with assay buffer supplied in the ELISA kit. The ELISA was run according to manufacturer’s protocol, and all samples were run in duplicates. Mean ± SEM intra-assay coefficient of variability (CV) for the assays was 7.75 ± 0.95.

### Statistical analysis

All data were tested for normality by a Shapiro–Wilk test. Water cortisol concentrations were corrected by subtracting average cortisol concentrations in blank controls and consistency in cortisol responsiveness between consecutive stress tests was tested by linear regression. Correlation was determined by a Pearson r test in case of a normal distribution and by a Spearman rank test in case of a non-normal distribution. Differences in cortisol response and body weights between females and males were tested by a two-tailed unpaired t-test followed by an F test to compare variances. Welch’s correction for unequal variances was applied when appropriate. Significance was accepted at *p* < 0.05. All values are given as mean ± SEM. All statistical analyses were performed in GraphPad Prism 7.0. (GraphPad Software, San Diego, CA, USA).

## Results

### Cortisol-response to stress is variable between, but consistent within individual zebrafish

For all fish across all tests performed in this study, post stress whole-body cortisol levels ranged between 14.9 and 100.6 ng g^-1^ fish, with an average of 44.9 ng g^-1^. That is similar to or higher than post stress whole-body cortisol levels previously reported for this species^22,51^. Cortisol levels were also markedly higher than resting whole-body cortisol levels (4–5 ng g^-1^) reported for zebrafish^22,51^.

To test for consistency in post-stress cortisol production, holding water from individual zebrafish was collected three times following three separate stress tests on the same individuals. Moreover, a fourth stress test was conducted to investigate consistency between water cortisol and whole-body cortisol measurements.

**Figure 2:**
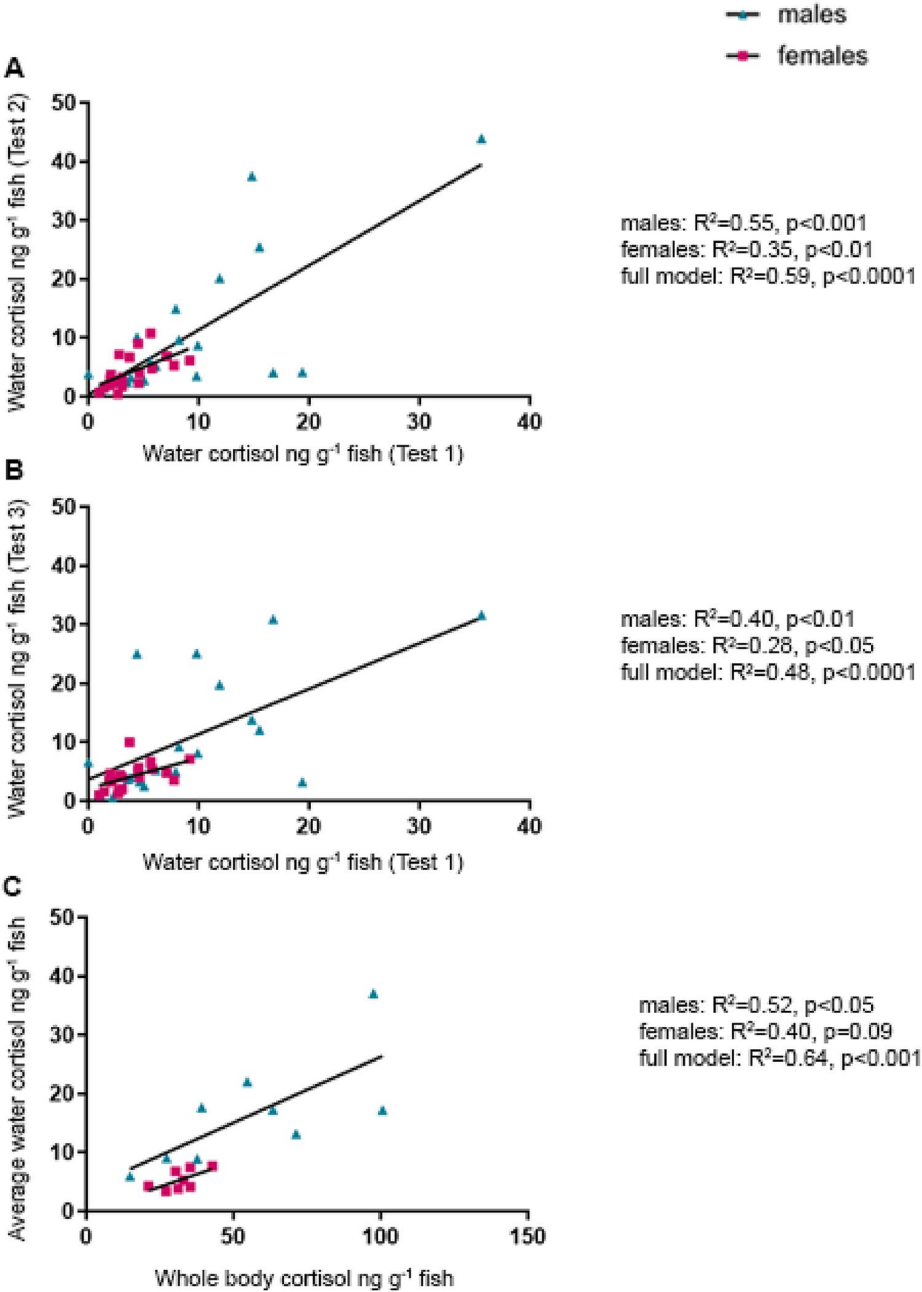
Cortisol-response to stress is variable between, but consistent within individual zebrafish (*Danio rerio*). Consistency and validity of post-stress water cortisol levels following acute stress was assessed by comparing water-borne cortisol levels following three consecutive stress tests (**A**, **B**) and mean water cortisol levels to whole body cortisol following a fourth stress test (**C**) in males (blue triangles) and females (pink squares). Consistency in cortisol responsiveness was tested by linear regression and correlation by a Pearson r test in case of a normal distribution and by a Spearman rank test in case of a non-normal distribution.

**Figure 3:**
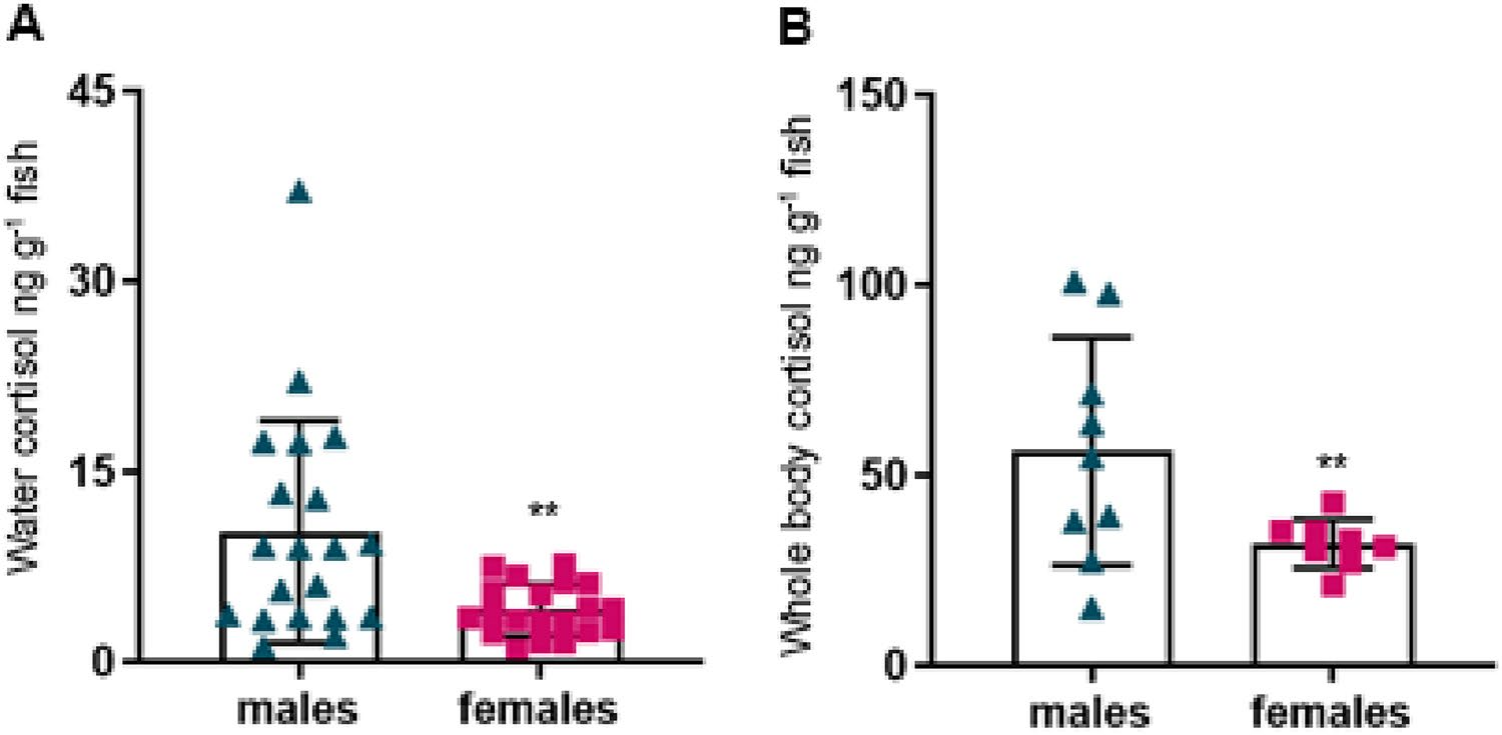
Cortisol levels is higher in males compared to female zebrafish. The magnitude in post-stress cortisol levels measured in (**A**) holding-water and (**B**) whole-body were lower for females compared to males. Statistical differences were tested by two-tailed un-paired t-tests. **p < 0.01 males versus females.

There were significant positive correlations in the measured cortisol levels between the separate tests for both male and female zebrafish (Fig. 2). More precisely, cortisol levels in the holding water from stress test 1 was a predictor of water cortisol levels in stress tests 2 (males: R^2^ = 0.55, *p* < 0.001, females: R^2^ = 0.35, *p* < 0.01, full model: R^2^ = 0.59, *p* < 0.0001, Fig. 2A) and 3 (males: R^2^ = 0.40, *p* < 0.01, females: R^2^ = 0.28, *p* < 0.05, full model: R^2^ = 0.48, *p* < 0.0001, Fig. 2B). Of note, positive correlations between holding water from the different stress tests were stronger for males than for females, mainly driven by one male that consistently responded to stress with considerably higher GC levels than other fish (36, 45 and 32 ng g^-1^ in tests 1, 2 and 3, respectively). When this individual was removed from the analyses, the associations became weaker, but remained statistically significant (test 1 vs test 2, males: R^2^ = 0.26, *p* = 0.03, test 1 vs test 3, males: R^2^ = 0.62, *p* < 0.001). Overall, average water cortisol levels from the three initial stress tests significantly predicted whole-body cortisol levels when both sexes were combined in the model, but whereas the association was significant in males it did not quite reach statistical significance in females (full model: R^2^ = 0.64, *p* < 0.001, males: R^2^ = 0.52, *p* < 0.05, females: R^2^ = 0.40, *p* = 0.09, Fig. 2C).

### Cortisol levels and between-individual variation is higher in males compared to females

Cortisol levels in both water (un-paired t-test: welch-corrected t = 3.07, df = 21.07, *p* < 0.01, Fig. 3A) and whole-body (un-paired t-test: welch-corrected t = 2.38, df = 8.82, *p* < 0.05, Fig. 3B) were markedly lower for females compared to males. Moreover, individual variation in post-stress cortisol levels measured in holding water (F = 18.27, DFn = 19, Dfd = 19, *p* < 0001) and whole-body (F = 21.93, DFn = 8, Dfd = 7, *p* < 001) was considerably higher in males compared to females (Fig. 3). There was no significant difference between male and female bodyweight (mean ± SEM for males: 0.4928 ± 0.04395 g, females: 0.6083 ± 0.05491 g, *p* = 0.11, t = 1.626, df = 48) or fork length (mean ± SEM for males: 3.233 ± 0.0418 cm, females: 3.132 ± 0.0474 cm, *p* = 0.12, t = 1.591, df = 27) for the fish tested in this study (data not shown).

## Discussion

In this study we observed that GC responsiveness to stress is a consistent individual trait in zebrafish. This new finding was determined using a technique that facilitated repeated, non-invasive sampling of the same individuals, followed by the extraction and quantification of GCs from the holding water of isolated stressed fish. We also confirmed a previously reported strong sexual dimorphism with regards to individual response patterns, *(sexual dimorphism)*, with female zebrafish responding to stress with lower levels of cortisol compared to male fish^22,44,45^. Our findings indicate consistency of the magnitude of post-stress GC production over several successive stress events in zebrafish, when surveyed under standardized conditions. Moreover, our study largely confirms that post-stress GC production and release can be reliably reflected by water-borne hormone levels^43^. However, since we found that the association between water-borne and whole-body cortisol levels was not as evident in female compared to male zebrafish, our study indicates that caution should be made when attempting to extrapolate GC production from water-borne cortisol levels if not controlling for fish sex.

A standardized sampling regime together with a sensitive and precise analytical method were key to the verification of our findings. Our data expand on two recent reviews and meta-analyses identifying considerable variation between studies regarding consistency of GC secretion^41,52^. Both meta-analyses found that GC repeatability measures vary between different taxa, with the highest repeatability found in amphibians. However, both reviews observe that an absolute majority of published studies on within-individual consistency of GC titers are conducted in birds (75–80% of studies), followed by mammals (15%) and poikilotherms (< 10%). The higher consistency was generally found in stress-induced versus resting GC levels, while pronounced differences in the magnitude of the physiological response to stress is a consistent observation between species. These and other studies suggest that there are fundamental evolutionary pressures which promote a diversity of persistent stress coping styles and response strategies among vertebrates^16,24,25,53^. The relative scarcity of studies in fish, amphibians, and reptiles highlights the need for further research in comparative models to clarify the evolutionary origin of this trend.

Indeed, in several teleost species, GC responsiveness to stress has been reported to correlate with other physiological and behavioral responses to stress^24,26,54^. For example, GC responsiveness is a highly consistent trait in rainbow trout (*Oncorhynchus mykiss*) and post stress GC levels accurately predict behavioral correlates of personality^23,55,56^, brain function, and neural plasticity^57–59^. For instance, GC responsiveness is generally a reliable predictor of social position, with low responding individuals usually becoming socially dominant^60^, but see Ruiz-Gomez et al.^61^ for an example of context-dependency in this pattern.

Previous studies have correlated singularly measured post-stress GC levels with behavioral indicators of stress coping style (as defined by Koolhaas et al.^19^) in zebrafish. For example, Tudorache et al.^32^ showed that proactive zebrafish larvae respond to stress with lower whole-body cortisol levels and a faster recovery back to baseline levels than reactive individuals. Similarly, Wong et al.^22^ found that post-stress GC responsiveness was lower in zebrafish selected to display low (LSB, presumably proactive) compared to high (HSB, presumably reactive) stationary behaviour. Although behavioral variation was not assessed in the current study, these previous observations along with the individual consistency in GC responsiveness reported here is consistent with the existence of stress coping styles in zebrafish. It is important to note that the fish tested in the current study came from an inbred lab strain of zebrafish and that the genetic variation in this lab population is likely to be considerably lower than in wild zebrafish. It is a reasonable assumption that GC responsiveness, being at least in part genetically based^62–64^ is likely to be even more variable in undomesticated fish populations.

Glucocorticoid hormones are major neuroendocrine integrators influencing vital biological processes including reproduction, growth, metabolism, immune function, cognition and emotion^2,65–67^. Hence, individual consistency in magnitude of the GC response to stress (as well as the possibility to accurately measure it) in zebrafish has several research implications. Firstly, in a fundamental perspective zebrafish emerge as a promising model to reveal the proximate and ultimate mechanisms underlying consistent phenotypic trait correlations. Secondly, zebrafish is already a favored animal model for high throughput research in many fields of biology, including stress-related diseases such as anxiety, depression, and cardiovascular disease^68–71^. Also with respect to infection biology, given the immune-modulating and neurodegenerative nature of GCs, stress reactivity is considered a likely predictor of disease vulnerability and trajectory^72,73^. Scientific questions often relate to how a particular pathogen or environmental condition affects disease vulnerability and progression. In many cases, any individual variation in severity of disease is regarded as noise and fail to appreciate such variation as a possible source of important biological information and knowledge. In this context, if correctly implemented in experimental design, non-invasive measurements of GC responsiveness may reveal whether individually different outcomes reflect inherent biological variation or unintended treatment variation.

Of note, if attempting to extrapolate GC production in the zebrafish body from water-borne cortisol levels it is important to consider potential sex-specific differences in the sensitivity of this method. We show that whereas water-borne GCs predict whole-body GC production in male zebrafish, the association was weaker and did not reach statistical significance in females (*p* = 0.09). We also show that in female zebrafish, both the magnitude and individual variation in post stress GC production is lower compared to in males. Thus, the method might not be sensitive enough to extrapolate whole body GC production from water-borne GCs in females. However, given the relatively low sample number in the current study (n = 8), we encourage future efforts to confirm or refute the value of this method also in studies using female zebrafish.

In summary, the current study confirms that water-borne GCs reliably reflect individually consistent variation in GC responsiveness at least in male zebrafish. This non-invasive approach has several advantages, both for research and animal welfare (reduction of animal numbers). Further, adopting this non-invasive method will result in the intact fish (and tissues) being available for subsequent behavioral, morphometric, physiological, or molecular analysis. Thus, zebrafish can provide unique insights into not only the molecular-genetic mechanisms underlying phenotypic variation, but also how such variation is constrained by selection^74^. Further validation of this method is provided by the observed sex differences with higher post-stress cortisol levels in male compared to female zebrafish^22,44,45^. The methods described here are also applicable to a wide range of other small-bodied aquatic vertebrates, a category which provides almost inexhaustible diversity for the study of stress responses as adaptive traits enabling the organism to cope with a changing environment.

## Data Availability

The datasets generated during and analyzed during the current study are available in the NMBU Open Research Data repository, [https://dataverse.no/dataset.xhtml?persistentId=doi:10.18710/HM1HXV].
